# IL-36γ Augments Host Defense and Immune Responses in Human Female Reproductive Tract Epithelial Cells

**DOI:** 10.3389/fmicb.2016.00955

**Published:** 2016-06-17

**Authors:** Sean M. Winkle, Andrea L. Throop, Melissa M. Herbst-Kralovetz

**Affiliations:** Department of Basic Medical Sciences, College of Medicine–Phoenix, University of Arizona, PhoenixAZ, USA

**Keywords:** IL-1 family, IL-36γ, human epithelial cells, antimicrobial peptides, innate immunity, IL-36 receptor, microbial products, inflammatory mediators

## Abstract

IL-36γ is a proinflamatory cytokine which belongs to the IL-1 family of cytokines. It is expressed in the skin and by epithelial cells (ECs) lining lung and gut tissue. We used human 3-D organotypic cells, that recapitulate either *in vivo* human vaginal or cervical tissue, to explore the possible role of IL-36γ in host defense against pathogens in the human female reproductive tract (FRT). EC were exposed to compounds derived from virus or bacterial sources and induction and regulation of IL-36γ and its receptor was determined. Polyinosinic-polycytidylic acid (poly I:C), flagellin, and synthetic lipoprotein (FSL-1) significantly induced expression of IL-36γ in a dose-dependent manner, and appeared to be TLR-dependent. Recombinant IL-36γ treatment resulted in self-amplification of IL-36γ and its receptor (IL-36R) via increased gene expression, and promoted other inflammatory signaling pathways. This is the first report to demonstrate that the IL-36 receptor and IL-36γ are present in the human FRT EC and that they are differentially induced by microbial products at this site. We conclude that IL-36γ is a driver for epithelial and immune activation following microbial insult and, as such, may play a critical role in host defense in the FRT.

## Introduction

The epithelium of the human FRT plays host to a wide variety of commensal bacteria and acts as a first responder to invading microbes and changes in the microbial milieu. The human FRT can be divided into two distinct regions that differ in cellular structure and function. The lower FRT epithelium is composed of the vagina and ectocervix and is lined with a stratified squamous epithelium built to resist physical and mechanical stress. The upper FRT epithelium is made up of a single layer of columnar EC and includes the endocervix, endometrium, fallopian tubes, and the uterus (Horne et al., 2008). Site-specific differences in the magnitude and pattern of immune response have been investigated in the vagina and endocervix (Quayle, 2002; Pudney et al., 2005; Herbst-Kralovetz et al., 2008).

To better understand immune function of the human FRT at these two sites, we investigated a newly characterized IL-1 family member identified to play a role in chronic inflammation and disease at other mucosal sites (Dunn et al., 2001; Gabay and Towne, 2015). The IL-36 family is a group of cytokines whose members are known for their role in mediating inflammation and host defense (Dunn et al., 2001; Gresnigt and van de Veerdonk, 2013; Gresnigt et al., 2013). While IL-36 family members have been shown to be expressed and secreted by the epithelium lining the lungs, gut and skin, this family, to our knowledge, has not previously been investigated in the human FRT (Chustz et al., 2011; Johnston et al., 2011; Gabay and Towne, 2015; Medina-Contreras et al., 2016). There are three isoforms that comprise the IL-36 family; IL-36α (IL-1F7), IL-36β (IL-1F8), and IL-36γ (IL-1F9) and these cytokines are expressed in a more tissue-restricted fashion relative to IL-1 (Dunn et al., 2001; Gresnigt and van de Veerdonk, 2013). Of the three isoforms, IL-36γ has the highest expression levels in damaged or infected epithelium that lines the skin and lungs suggesting that it plays a role in epithelial immune response (Berglof et al., 2003; Lian et al., 2012; Gabay and Towne, 2015). As tissue becomes diseased, IL-36γ promotes inflammation by binding to the IL-36 receptor (IL-36R) and stimulating the production of a number of cytokines, chemokines, adhesion molecules, antimicrobial peptides, and pro-inflammatory mediators (Towne et al., 2004; Vigne et al., 2011; Gresnigt and van de Veerdonk, 2013). Triggering IL-36R is necessary for IL-36γ to activate pathways that lead to induction of pro-inflammatory cytokine expression via NF-κB and MAPKs pathways (Chustz et al., 2011; Towne et al., 2011).

Vaginal and endocervical EC express pattern recognition receptors (PRRs) that can be triggered by sensing microbial products associated with bacteria and viruses resulting in an innate immune response (Herbst-Kralovetz et al., 2008). EC of the lower FRT respond to microbial products by secreting cytokines, chemokines, and antimicrobial peptides (Hjelm et al., 2010; Radtke et al., 2012; Doerflinger et al., 2014). Toll-like receptors (TLRs) expressed in FRT EC recognize microbe-associated molecular patterns (MAMPs) that correspond to highly conserved microbial products. Recent studies have shown that TLR agonists including polyinosinic-polycytidylic acid [poly (I:C); viral product] and flagellin (bacterial product) upregulate IL-36γ production in human skin keratinocytes, but have differential effects on IL-36γ secretion (Lian et al., 2012). Similar to IL-1β and IL-18, the IL-36 family members require processing to gain full bioactivity, the enzyme required is yet to be identified (Towne et al., 2011; Gabay and Towne, 2015). While both microbial products induce IL-36γ expression, only poly (I:C) has been shown to induce secretion (Lian et al., 2012). As FRT EC express TLRs 2, 3, 5, and 6 at the highest levels (Herbst-Kralovetz and Pyles, 2006), we tested poly (I:C; TLR3 agonist), flagellin (TLR5 agonist), and FSL-1 (bacterial product; TLR2/6 agonist) for induction and secretion of IL-36γ in the human FRT.

To investigate the function and regulation of IL-36γ in the FRT, we used our well-characterized 3-D human vaginal and endocervical EC models (Barrila et al., 2010; Hjelm et al., 2010; Radtke et al., 2012; Doerflinger et al., 2014). These models faithfully recapitulate many of the physiologic traits of FRT EC, including cellular architecture, adhesion, interaction, and immune function (Hjelm et al., 2010; Radtke et al., 2012; McGowin et al., 2013; Doerflinger et al., 2014). Furthermore, these two models can be used to evaluate site-specific differences in the magnitude and/or pattern of immune response at these sites. FRT EC models were exposed to microbial products to measure innate inflammatory responses in the FRT via induction and secretion of IL-36γ. In addition, we investigated the autocrine function of IL-36γ in these 3-D FRT human EC by treating with rIL-36γ. This is the first report to demonstrate that IL-36 receptor and IL-36γ are both expressed by ECs lining the human lower FRT and important in regulating host defense and immune responses in a TLR and site-specific manner.

## Materials and Methods

### Culturing 3-D Human Vaginal and Endocervical Epithelial Cells

Human endocervical (A2) ECs were added to a suspension of collagen-coated dextran microcarrier beads (Sigma-Aldrich, St. Louis, MO, USA) in KSFM (Life Technologies, Grand Island, NY, USA) supplemented with 5 ng/ml human recombinant epidermal growth factor, 500 μg/ml bovine pituitary extract, 44 μg/ml CaCl_2_ (Sigma-Aldrich), and 100 μg/ml primocin (InvivoGen, San Diego, CA, USA) as previously described (Radtke et al., 2012). Human vaginal (V19I) EC were added to a suspension of collagen-coated dextran microcarrier beads in a 1:1 mixture of supplemented KSFM and EpiLife medium (Life Technologies) as previously described (Hjelm et al., 2010). Cell and bead suspensions were transferred into a slow turning lateral vessel bioreactor and incubated at 37°C as previously described (Hjelm et al., 2010; Radtke and Herbst-Kralovetz, 2012; Radtke et al., 2012). After 28 days of growth, differentiated endocervical and vaginal aggregates were quantified using a Countess machine (Life Technologies) and viability was measured by trypan blue exclusion.

### Human Cervical and Vaginal Epithelial Tissue Total RNA and Protein

Human cervical and vaginal total protein samples and human cervical and vaginal total RNA samples were purchased from BioChain (Newark, CA, USA). According to BioChain, human vaginal total protein and total RNA samples were acquired from a post-menopausal Caucasian female. Human cervical total protein samples were acquired from a pre-menopausal Asian female and human cervical total RNA samples were acquired from a pre-menopausal Caucasian female (BioChain). As per Biochain, all analyzed tissues were determined to be non-diseased per the clinical report and were screened by serology for HIV, HBV, HCV, and HTLV. cDNA was synthesized from total RNA (1 μg) cDNA by reverse transcription (iScript cDNA Synthesis Kit, Bio-Rad, Hercules, CA, USA) prior to qRT-PCR assays.

### TLR Agonist and Cytokine Treatment

For all experimental treatments, 3-D aggregates were transferred into 24-well plates (1 × 10^5^–5 × 10^5^ cells per ml). Both 3-D vaginal EC and 3-D endocervical EC were treated with poly (I:C; InvivoGen) at 1, 25, and 100 μg/ml, flagellin (InvivoGen) at 0.5 and 5 μg/ml, FSL-1 (InvivoGen) at 0.01 and 0.1 μg/ml, or recombinant IL-36γ (PeproTech, Rocky Hill, NJ, USA) at 1, 10, 100, and 500 ng/ml for 24 h at 37°C. Additional wells were treated with sterile Dulbecco’s PBS to provide a negative control. Following cell culture treatment, supernatants were harvested and stored at -20°C until analysis. Cell pellets were stored at -20°C in lysis buffer for RNA or protein extraction.

### Protein Extraction and Quantification

For protein extraction, aggregates were pelleted and stored at -20°C in 1 ml of RIPA buffer [150 mM NaCl (Thermo Fisher Scientific), 50 mM Tris-HCL pH 8.0 (Life Technologies), 1 mM EDTA (Life Technologies), 1% NP-40 (Sigma-Aldrich), 1% sodium deoxycholate (Sigma-Aldrich), 0.1% SDS (Sigma-Aldrich), 1 mM PMSF (Sigma-Aldrich), and 5 μg/ml leupeptin (Sigma-Aldrich)]. Frozen cell pellets were thawed on ice. Once thawed, cells were incubated for 10 min on ice with intermittent mixing by inversion. Next, cells were sheared with a 23-gage needle connected to a 1 ml syringe. After incubating on ice for 20 min, cells were pelleted at 10,000 × *g* and supernatant was removed. A Bradford assay was performed in a microtiter plate to determine total protein concentration of the extracted cell pellet and culture supernatant. Absorbance was read at 595 nm on a Biotek ELx800 Microplate Reader (BioTek, Winooski, VT, USA) and experimental values were compared to the calculated standard curve to acquire total protein concentration.

### ELISA Quantification of Intracellular and Secreted Human IL-36γ

High binding microtiter plates (Corning, Tewksbury, MA, USA) were coated with 50 μl/well of monoclonal rat anti-human IL-36γ antibody (R&D Systems, Minneapolis, MN, USA) at 2 μg/ml in PBS and incubated overnight at 4°C. The microtiter plates were washed three times with PBS-0.05%Tween-20 (PBST) then 50 μl of twofold serial dilutions of recombinant human IL-36γ (rIL-36γ, PeproTech) was added in duplicate to generate a standard curve. Experimental samples were added to each well in duplicate and all samples were incubated for 2 h at 37°C. The microtiter plates were then washed three times with PBST then biotinylated polyclonal goat anti-human IL-36γ detection antibody (R&D Systems) diluted at 2 μg/ml with 1% skim milk in PBST was added to each well and incubated for 2 h at 37°C. Following the incubation, the plates were washed with PBST three times. After washing, 50 μl streptavidin-HRP (R&D Systems) was added at a 1:250 dilution in PBST to each well and incubated for 1 h at 37°C. The plates were washed three times with PBST and then developed by addition of 50 μl tetramethylbenzidine substrate solution (Thermo Fisher Scientific, Waltham, MA, USA) to each well and incubated in the dark for up to 30 min at room temperature. The colorimetric reaction was stopped by addition of 50 μl/well of 1 M phosphoric acid and absorbance read at 450 nm on a Biotek ELx800 Microplate Reader (BioTek). Results were reported in fold as compared to PBS treated cell extracts.

### Human IL-36γ Western Blot Analysis

Cell culture supernatants and cell pellet extracts were boiled for 10 min in 2× SDS buffer (6% SDS, 25 mM Tris-HCL pH 6.5, 10% glycerol, 0.1 M DTT, 20 μg/ml bromophenol blue). Total protein (30 μg) was loaded into 4–15% polyacrylamide Mini-PROTEAN TGX precast gels (Bio-Rad). After proteins were separated by SDS-PAGE, gels were transferred to polyvinylidene diflouride membranes (Life Technologies) using a dry blotting system (iBlot, Life Technologies). Levels of IL-36γ were determined using biotinylated goat anti-human IL-36γ diluted to 4 μg/ml in PBST with 1% dry milk, followed by streptavidin-HRP diluted 1:250 (R&D Systems). Levels of β-tubulin were examined using mouse anti-β-tubulin (Santa Cruz, Biotechnology, Dallas, TX, USA) diluted 1:1000 with horseradish peroxidase labeled goat anti-mouse (Santa Cruz Biotechnology) as a secondary antibody. Membranes were developed using ECL substrate (Life Technologies).

### Quantification of Human Cytokines and Chemokines by Multiplex Analysis

Supernatants from 3-D vaginal and endocervical EC aggregates treated with rIL-36γ as described above were collected cytokine secretion levels were quantified. Cytokine concentrations were determined using a custom four-plex human cytokine kit containing IL-1B, IL-6, CCL20, and TNFα (EMD Millipore, Billerica, MA, USA) using the manufacturer’s protocol. The data were collected using a Bio-Plex 200 System with Bio-Plex 5.0 Manager software (Bio-Rad).

### RNA Extraction and Quantitative Real-Time PCR Analysis

RNA was extracted from 3-D endocervical and 3-D vaginal EC using the Qiagen RNeasy kit following the manufacturer’s instructions (Qiagen, Valencia, CA, USA). cDNA was synthesized from 1 μg RNA by reverse transcription (iScript cDNA Synthesis Kit, Bio-Rad) and analyzed by qRT-PCR. qRT-PCR was performed with an Applied Biosystems 7500 Fast Real Time PCR System (Life Technologies) using customized primers purchased from IDT (Integrated DNA Technologies, Coralville, IA, USA) and iTAQ Universal SYBR Green Supermix (Bio-Rad). The following primers were used in this study: IL-1β forward, 5′-ACAGATGAAGTGCTCCTTCCA-3′ and reverse 5′-GTCGGAGATTCGTAGCTGGAT-3′ (Stordeur et al., 2002), HE4 forward 5′-CGGCTTCACCCTAGTCTCAG-3′ and reverse 5′-AAAGGGAGAAGCTGTGGTCA-3′ (Drapkin et al., 2005), IL-36γ forward 5′-CCCAGTCACTGTTGCTGTTA-3′ and reverse 5′-CAGTCTTGGCACGGTAGAAA-3′, IL-36R forward 5′-GCTGGAGTGTCCACAGCATA-3′ and reverse 5′-GCGATAAGCCCTCCTATCAA-3′ (Mutamba et al., 2012). IL-6, IL-8, HBD2, HBD4, and SLPI primers were previously described (Radtke et al., 2012; Doerflinger et al., 2014). Relative transcript levels were determined using a GAPDH housekeeping gene transcript and are reported as fold relative to negative control unless otherwise noted.

### Statistical Analysis

All of the data for this study was generated using three independent batches of 3-D endocervical or vaginal EC aggregates in three independent experiments, and an average of all three of these experimental results are presented. An unpaired two-tailed Student *t*-test with Welch’s correction was performed to determine statistical significance. GraphPad Prism v5.0 software was used for statistical analysis (GraphPad, San Diego, CA, USA). Levels of significance are reported as follows; ˆ*P* < 0.05; ^∗^*P* < 0.01; ^∗∗^*P* < 0.001.

## Results

### Human Tissue and 3-D Vaginal and Endocervical EC Express IL-36γ and IL-36R

**FIGURE 1:**
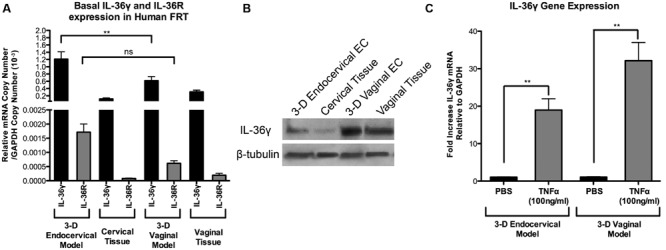
**Human 3-D vaginal and cervical epithelial cells and tissue express IL-36γ and IL-36R. (A)** Expression of IL-36γ and IL-36R in 3-D human vaginal and endocervical cell cultures, and human vaginal and cervical tissue. cDNA from cell cultures and tissue were analyzed by qRT-PCR and mRNA copy numbers were normalized against GAPDH. **(B)** Cell lysates from 3-D vaginal and endocervical cell cultures, vaginal tissue and cervical tissue were evaluated by Western blot for expression of IL-36γ. β-tubulin was used as a loading control. 30 μg total protein from each lysate was loaded. **(C)** TNFα (100 ng/ml) treated 3-D cultures of vaginal and endocervical epithelial cells expressed IL-36γ. mRNA levels were normalized against GAPDH and expression of IL-36γ was reported as fold change compared to PBS treated samples. Data are shown as mean ± SD from three independent experiments each performed in duplicate. ˆ*P* < 0.05; ^∗^*P* < 0.01; ^∗∗^*P* < 0.001; unpaired two-tailed Student *t*-test with Welch’s correction.

IL-36γ has been previously shown to be expressed in skin and ECs lining mucosal tissue (Jensen, 2010; Chustz et al., 2011; Gabay and Towne, 2015), however, this cytokine has not been shown to be expressed previously in the FRT. Human IL-36γ levels were measured in both 3-D vaginal and endocervical EC by qRT-PCR analysis. Untreated 3-D vaginal and endocervical EC, and human cervical and vaginal tissue were analyzed by qRT-PCR assays targeting IL-36γ and IL-36R (**Figure 1A**). Basal expression of IL-36γ was significantly (*P* < 0.001) higher in 3-D endocervical EC compared to 3-D vaginal EC. Human 3-D vaginal and endocervical EC, as well as, human vaginal and cervical tissue expressed IL-36γ at levels much higher than IL-36R (**Figure 1A**). This is the first report to characterize IL-36R and IL-36γ in the EC lining the human FRT, furthermore, these results demonstrate our model’s ability to serve as a tool to further study IL-36 function.

Western blots were performed to confirm production/synthesis of human IL-36γ in EC of the FRT. Cell lysates from human 3-D vaginal and endocervical EC models and cell lysates from human cervical and vaginal tissue, were analyzed by Western blot (**Figure 1B**). We found that IL-36γ was constitutively produced in human 3-D vaginal and endocervical EC and vaginal and endocervical tissue. Basal synthesis of IL-36R was lower than the detection limits of Western blot analysis (data not shown) in both PBS treated and TLR agonist stimulated FRT EC cells. TNFα, a cytokine previously shown to induce IL-36γ (Friedrich et al., 2014), was used as a positive control to test if IL-36γ is inducible in the FRT EC. Three-D vaginal and endocervical EC were treated with TNFα for 24 h. Following TNFα treatment, IL-36γ gene expression levels in 3-D vaginal and endocervical EC were significantly (*P* < 0.001) increased 32-fold, and 19.6-fold respectively (**Figure 1C**). The 3-D vaginal EC and tissue exhibited higher baseline levels of IL-36γ and IL-36R relative to 3-D endocervical EC and tissue and TNFα treatment induced higher levels of IL-36γ expression in 3-D vaginal EC relative to the endocervical EC.

### Microbial Products Differentially Induce IL-36γ Expression and Secretion in EC of the Human FRT

**FIGURE 2:**
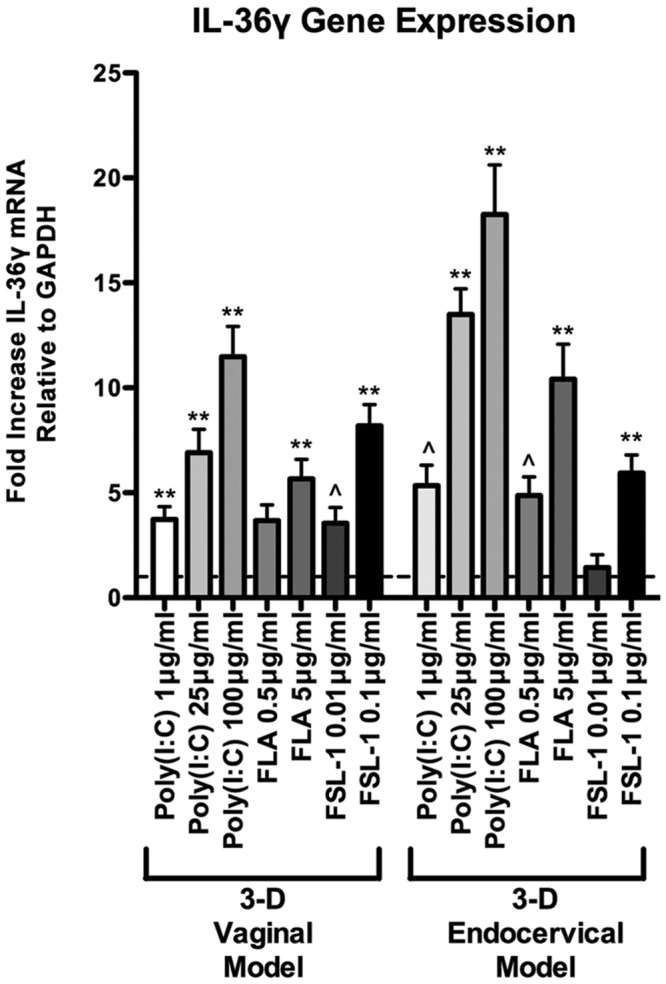
**Differential IL-36γ induction in 3-D human vaginal and endocervical epithelial cells treated with microbial products.** Gene expression analysis of 3-D vaginal and endocervical EC. Cell cultures were exposed to microbial products [poly (I:C); 1, 25, and 100 μg/ml, flagellin (FLA); 0.5 and 5 μg/ml, and fibroblast stimulating lipopeptide-1 (FSL-1); 0.01 and 0.1 μg/ml] for 24 h. Expression of IL-36γ was determined by qRT-PCR and reported relative to GAPDH as fold change compared to PBS treated samples. Horizontal dashed line indicates IL-36γ expression in PBS treated cells. Data are shown as mean ± SD from three independent experiments each performed in duplicate. ˆ*P* < 0.05; ^∗^*P* < 0.01; ^∗∗^*P* < 0.001; unpaired two-tailed Student *t*-test with Welch’s correction.

Microbial products elicit potent immune responses in EC of the FRT through ligation with TLRs. We have previously shown that TLR agonists: poly(I:C; viral product and TLR3 agonist), flagellin (bacterial product and TLR5 agonist), and FSL-1 (bacterial product and TLR2/6 agonist) induce cytokine expression and secretion in primary and immortalized vaginal and endocervical EC grown as monolayers and in 3-D (Herbst-Kralovetz et al., 2008; Hjelm et al., 2010; Radtke et al., 2012; Doerflinger et al., 2014). These studies have also shown that TLRs 2, 3, 5, and 6 are abundantly expressed in these cell lines and these TLRs remain functional and responsive when cultured under rotating conditions (Herbst-Kralovetz et al., 2008; Hjelm et al., 2010; Radtke and Herbst-Kralovetz, 2012; Radtke et al., 2012). Maximal cytokine induction was detected 24 h after treatment of microbial products in these studies, hence we focused on that time point herein (Herbst-Kralovetz et al., 2008). Human 3-D vaginal and endocervical EC were treated with a panel of microbial products for 24 h, and IL-36γ expression levels were quantified by qRT-PCR analysis (**Figure 2**). Following poly (I:C), FSL-1, and flagellin treatment did not result in cellular adherence to beads as observed by light microscopy and cellular viability was not decreased by more than 10% as measured by trypan blue exclusion (data not shown). IL-36γ expression increased following treatment with each microbial product in a concentration dependent manner. We observed that poly (I:C) induced the highest amount of IL-36γ gene expression in both cell types (**Figure 2**). In 3-D vaginal EC IL-36γ expression was induced by treatment with poly (I:C; 100 μg/ml), flagellin (5 μg/ml), and FSL-1 (0.1 μg/ml) by 10.2-, 5.1-, and 8.9-fold, respectively. Endocervical EC treated with poly (I:C), flagellin, and FSL-1 also resulted in significant increases (*P* < 0.001) in IL-36γ expression levels at 20.2-, 11.7-, and 7-fold, respectively. Poly (I:C) and flagellin induced higher levels of IL-36γ expression in 3-D endocervical EC relative to vaginal EC (*P* < 0.001).

**FIGURE 3:**
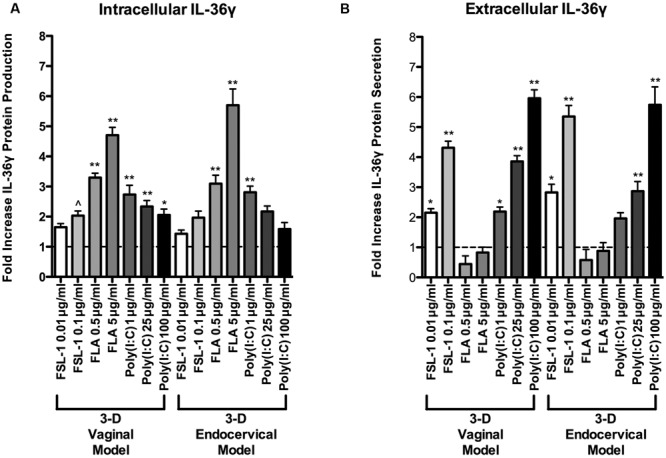
**Extracellular and intracellular localization of IL-36γ in epithelial cells of the human female reproductive tract following treatment with microbial products.** Three-D vaginal and endocervical EC were treated with PBS, flagellin (FLA; 5 or 0.5 μg ml^-1^), FSL-1 (0.1 or 0.01 μg ml^-1^), or poly (I:C; 100, 25, or 1 μg ml^-1^) for 24 h. **(A)** Cell culture lysates and **(B)** supernatants were assayed by ELISA to quantify IL-36γ production and secretion. Levels of IL-36γ were determined and reported as fold change relative to PBS treated samples. Horizontal dashed line indicates IL-36γ expression in PBS treated cells. Data are shown as mean ± SD from three independent experiments each performed in duplicate. ˆ*P* < 0.05; ^∗^*P* < 0.01; ^∗∗^*P* < 0.001; unpaired 2-tailed Student *t*-test with Welch’s correction.

Since IL-36γ mRNA levels increased in a dose dependent manner, we performed an IL-36γ targeted ELISA to quantify protein levels following treatment with these microbial products. After treatment with the poly (I:C), flagellin, FSL-1, and PBS, cell lysates and cellular supernatants were collected and assayed for IL-36γ by ELISA. Poly (I:C), flagellin, and FSL-1 treatments induced IL-36γ production in a dose dependent manner in both 3-D vaginal and 3-D endocervical EC when compared to PBS treated controls (**Figure 3B**). All of the microbial products tested induced an increase in IL-36γ mRNA in 3-D FRT EC, however, the magnitude of protein production and secretion levels were microbial product specific. Treatment with higher concentrations of poly (I:C) resulted in higher levels of secreted IL-36γ in 3-D vaginal and endocervical EC (**Figures 3A,B**) when measured by ELISA. Flagellin induced a significant increase (*P* < 0.01) in intracellular IL-36γ production in vaginal and endocervical (*P* < 0.01) EC. Poly (I:C) induced significant levels of IL-36γ secretion by vaginal (1 μg/ml; *P* < 0.01, 25 μg/ml; *P* < 0.001, and 100 μg/ml; *P* < 0.001) and endocervical (1 μg/ml; [not significant (NS)] 25 μg/ml; *P* < 0.001, and 100 μg/ml; *P* < 0.001) EC. FSL-1 induction of intracellular IL-36γ production significantly increased secretion of IL-36γ in both vaginal (0.01 μg/ml; *P* < 0.01, 0.01 μg/ml; *P* < 0.001) and endocervical (0.01 μg/ml; *P* < 0.01, 0.01 μg/ml; *P* < 0.001) EC. Less IL-36γ secretion occurred following treatment with flagellin relative to control treated cells.

IL-36γ probed Western blots were performed to verify ELISA results. FRT EC were treated with poly (I:C), flagellin, and FSL-1 in increasing concentrations for 24 h. Both the supernatant and extracted cell pellets of TLR agonist treated FRT EC were analyzed to measure intracellular and secreted IL-36γ levels. Patterns of IL-36γ protein synthesis detected by Western blot mirrored the quantitative data generated by ELISA, supporting the protein production profiles of FRT EC (Supplementary Figure S1). Poly (I:C) and FSL-1 treatment resulted in the highest induction of secreted IL-36γ levels relative to untreated wells by ELISA and Western blot, whereas flagellin treatment induced significantly higher intracellular levels of IL-36γ (**Figure 3** and Supplementary Figure S1). As such, there was an inverse relationship between IL-36γ induction by these microbial products.

### Recombinant IL-36γ Induces an Autocrine IL-36γ Loop in Human 3-D Vaginal and Endocervical EC

**FIGURE 4:**
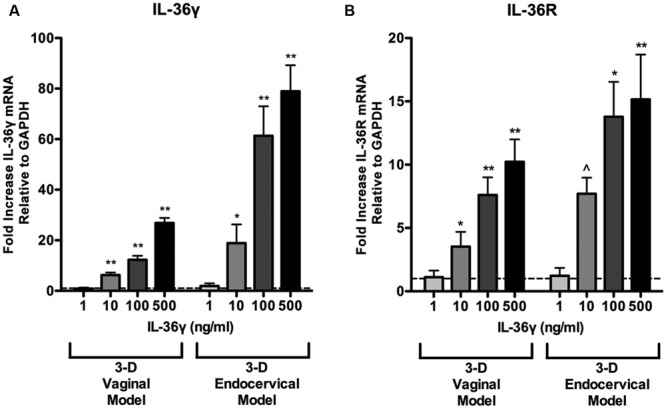
**Induction of IL-36γ and IL-36R expression following recombinant IL-36γ treatment.** 3-D vaginal and endocervical EC were treated with increasing doses of recombinant IL-36γ (1, 10, 100, and 500 ng/ml) for 24 h. **(A)** IL-36γ and **(B)** IL-36R expression was determined by qRT-PCR and reported relative to GAPDH as fold change compared to PBS treated samples. Horizontal dashed line indicates IL-36γ expression in PBS treated cells. Data are shown as mean ± SD from three independent experiments each performed in duplicate. ˆ*P* < 0.05; ^∗^*P* < 0.01; ^∗∗^*P* < 0.001; unpaired two-tailed Student *t*-test with Welch’s correction.

IL-36γ has been shown to induce expression of itself, thereby exhibiting an autocrine signaling loop in keratinocytes (Lian et al., 2012). Three-D vaginal and endocervical EC were treated with increasing doses of rIL-36γ (1, 10, 100, and 500 ng/ml) for 24 h. qRT-PCR assays targeting IL-36γ and IL-36R was performed following treatment with rIL-36γ. We observed a significant increase in IL-36γ gene expression in both 3-D vaginal (10 ng/ml; *P* < 0.001, 100 ng/ml; *P* < 0.001, 500 ng/ml; *P* < 0.001) and endocervical EC (10 ng/ml; *P* < 0.01, 100 ng/ml; *P* < 0.001, 500 ng/ml *P* < 0.001; **Figure 4A**). A similar pattern in IL-36R expression in 3-D vaginal [10 ng/ml; 4.2-fold (*P* < 0.01), 100 ng/ml; 7.9-fold (*P* < 0.001), 500 ng/ml; 10.4-fold (*P* < 0.001)] and endocervical EC [10 ng/ml; 8.3-fold (*P* < 0.05), 100 ng/ml; 13.9-fold (*P* < 0.01), 500 ng/ml; 15.7-fold (*P* < 0.001; **Figure 4B**)] was observed, albeit at lower levels. The fold increases in both IL-36γ and IL-36R were dependent on rIL-36γ treatment concentration and expression levels were higher in the 3-D endocervical EC compared to the vaginal EC.

### Pro-inflammatory Cytokine and Antimicrobial Peptide Production in 3-D FRT EC is Increased Following Recombinant IL-36γ Treatment

**FIGURE 5:**
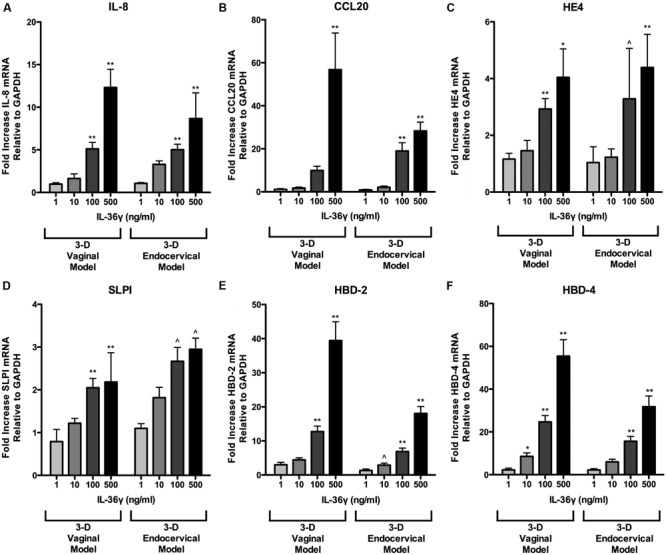
**Recombinant IL-36γ treatment induces antimicrobial peptide expression in 3-D human vaginal and endocervical epithelial cells.** 3-D vaginal and endocervical cell cultures were treated with increasing doses of recombinant IL-36γ (1, 10, 100, or 500 ng/ml) for 24 h. Relative expression levels of **(A)** IL-8, **(B)** CCL20, **(C)** HE4, **(D)** SLPI, **(E)** HBD-2, and **(F)** HBD-4 were determined by qRT-PCR and reported relative to GAPDH as fold change compared to PBS treated cells Data are shown as mean ± SD from three independent experiments each performed in duplicate. ˆ*P* < 0.05; ^∗^*P* < 0.01; ^∗∗^*P* < 0.001; unpaired two-tailed Student *t*-test with Welch’s correction.

**FIGURE 6:**
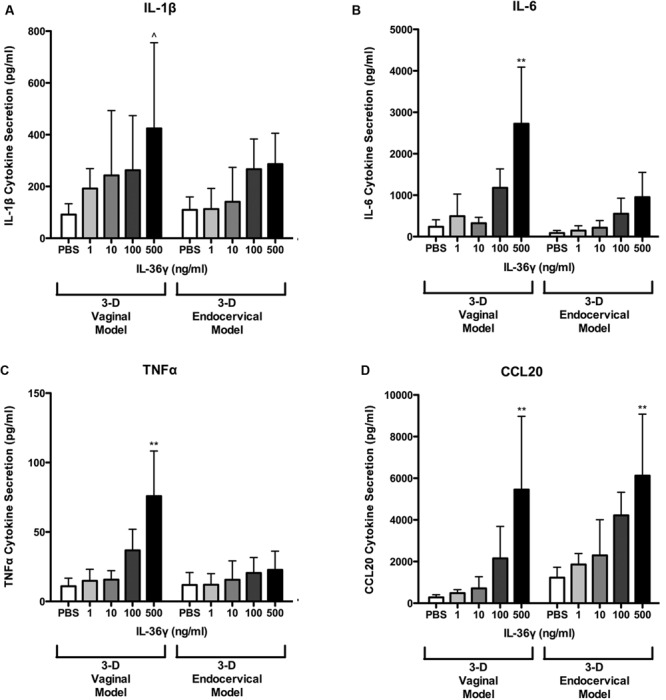
**3-D human vaginal and endocervical epithelial cells secrete increased levels of pro-inflammatory cytokines and chemokines in response to treatment with recombinant IL-36γ.** 3-D vaginal and endocervical cell cultures were treated with PBS or increasing doses of recombinant IL-36γ (1, 10, 100, or 500 ng/ml) for 24 h. Expression levels of **(A)** IL-1β, **(B)** IL-6, **(C)** TNFα, and chemokine **(D)** CCL20 were determined by multiplex analysis and reported in pg/ml. Data are shown as mean ± SD from three independent experiments each performed in duplicate. ˆ*P* < 0.05; ^∗^*P* < 0.01; ^∗∗^*P* < 0.001; unpaired two-tailed Student *t*-test with Welch’s correction.

We have shown that microbial products induce IL-36γ and influence its secretion. To examine the role IL-36γ plays in triggering inflammatory signaling pathways in FRT EC and to determine if it is concentration dependent, we treated 3-D endocervical and vaginal EC with rIL-36γ. Treatment with rIL-36γ induced antimicrobial peptide, pro-inflammatory cytokine, and chemokine production in a dose dependent manner (**Figures 5** and **6**). IL-8 (**Figure 5A**) mRNA levels were significantly increased (*P* < 0.001) in both cell models following 100 and 500 ng/ml rIL-36γ treatments. rIL-36γ treatment (500 ng/ml) resulted in a significant difference (*P* < 0.001) in CCL20 production (**Figure 5B**) and 3-D vaginal EC CCL20 levels were significantly higher (*P* < 0.001) at 500 ng/ml compared to the 3-D endocervical EC. Antimicrobial peptides including HE4 (**Figure 5C**), SLPI (**Figure 5D**), HBD-2 (**Figure 5E**), and HBD-4 (**Figure 5F**) were significantly increased in a dose dependent manner following rIL-36γ treatments. Treatment with rIL-36γ at 100 and 500 ng/ml resulted in significant increases in HE4 in 3-D vaginal (*P* < 0.001 and *P* < 0.01) and endocervical (*P* < 0.05 and *P* < 0.001). SLPI mRNA levels increased at similar magnitudes following rIL-36γ treatments at 100 and 500 ng/ml in 3-D vaginal (*P* < 0.001) and endocervical (*P* < 0.05) EC.

Of these AMP, two notable site-specific differences were observed in the induction HBD-2 and HBD-4 in 3-D FRT EC following treatment with100 and 500 ng/ml of rIL-36γ. Fold values for 3-D vaginal cells were higher for HBD-2 and HBD-4 relative to endocervical EC. HBD-2 increased 12.7-fold (*P* < 0.001) and 39.4-fold (*P* < 0.001) and HBD-4 mRNA was increased 24.6-fold (*P* < 0.001) and 55.4-fold (*P* < 0.001) in 3-D vaginal EC, respectively. rIL-36γ treated 3-D endocervical EC increased 6.9- and 18-fold at 100 and 500 ng/ml, respectively, for HBD-2 at 15.6-fold (*P* < 0.001) and 31.8-fold (*P* < 0.001) for the HBD-4 gene. HBD 2 and HBD-4 expression was significantly higher in 3-D vaginal EC than 3-D endocervical EC following rIL-36γ treatments at 100 and 500 ng/ml (*P* < 0.05 and *P* < 0.01 respectively). SLPI and HE4, were the only AMP assayed that resulted in more robust response in endocervical EC when compared to vaginal EC, but these site specific differences in expression were not significant. IL-8 gene expression levels were similar in both cell types. CCL20 expression levels varied in the two cell types depending on the concentration of rIL-36γ.

We have shown that rIL-36γ increases gene expression of chemokines and AMP. To quantify levels of additional proinflammatory cytokines and chemokines we assayed cell supernatants with a multiplex bead-based immunodetection assay. Secretion of IL-1β, IL-6, CCL20, and TNFα was induced in a dose dependent fashion following 24 h treatments with rIL-36γ (**Figure 6**). CCL20 secretion was significantly (*P* < 0.01) increased following treatment with rIL-36γ at 100 and 500 ng/ml in both 3-D vaginal and endocervical EC. IL-1β secretion was significantly (*P* < 0.01) increased following rIL-36γ treatments at 500 ng/ml in 3-D vaginal EC. rIL-36γ treatments at 100 and 500 ng/ml resulted in significant increases in IL-1β secretion (*P* < 0.05) in 3-D endocervical EC as well. Secretion of IL-6 was significantly (*P* < 0.01) increased in 3-D vaginal EC following rIL-36γ treatments of 100 and 500 ng/ml. IL-6 and TNFα secretion was induced in 3-D endocervical EC, however, the magnitude of secretion was higher in 3-D vaginal EC. Secreted IL-6 increased from 86.48 to 552.9 pg/ml (*NS*) for 100 ng/ml rIL-36γ and 951.5 pg/ml (*P* < 0.01) for 500 ng/ml rIL-36γ. TNFα levels from 3-D endocervical EC increased only slightly following rIL-36γ treatments and the increase was not significant. Overall, pro-inflammatory cytokines, chemokines, and AMP were significantly induced in 3-D vaginal and cervical EC following treatment with rIL-36γ. The pattern of cytokine and AMP induction was similar for vaginal and cervical cells and was also dose-dependent, but the magnitude was higher in the 3-D vaginal EC model relative to the 3-D cervical model for most immune mediators evaluated.

## Discussion

This is the first report demonstrating a role for IL-36γ in host defense and amplification of the mucosal immune response by ECs lining the FRT. We showed that in the lower FRT, IL-36γ stimulates the innate immune response by induction of pro-inflammatory cytokines, chemokines, and AMP, thereby promoting mucosal inflammation. This study provides the foundation for future studies that aim to elucidate the mechanisms of IL-36 family members in the FRT during homeostasis, STI, disease pathogenesis, and reproductive sequelae.

Recent literature on IL-36γ has been primarily focused on chronic inflammatory processes that include psoriasis and chronic obstructive pulmonary disease (COPD), where IL-36γ was found to be abundantly expressed in the skin and the mucosal ECs of the lung (Li et al., 2014; Gabay and Towne, 2015) and result in sustained inflammation (Chustz et al., 2011; Gabay and Towne, 2015). However, there have been limited studies investigating the role of IL-36γ in host defense and infection at mucosal sites (Vos et al., 2005; Gresnigt et al., 2013; Segueni et al., 2015).

Herein we report that IL-36γ is expressed in human cervical and vaginal ECs and tissue. A limitation to the study evaluating IL-36γ and IL-36R in vaginal tissue, is the lack of availability of premenopausal non-diseased vaginal tissue. However, our data demonstrated that premenopausal cervical tissue and post-menopausal vaginal tissue both express IL-36γ and IL-36R. Future studies will be required to determine the impact of sex hormones on this cytokine and associated receptor expression. Using our well-characterized human 3-D cervical and vaginal EC models we demonstrate that these cells respond to rIL-36γ exposure in a site-specific manner. Overall, 3-D vaginal EC expressed and secreted higher levels of these innate immune mediators following rIL-36γ treatments relative to 3-D endocervical EC, however, this response did not seem to correspond to IL-36R expression (**Figure 1**).

IL-36γ increases and prolongs inflammation through interaction with TNFα, a known inducer and inductee of IL-36γ (Towne and Sims, 2012). TNFα is expressed in excess in psoriatic lesions (Cuchacovich et al., 2008) leading to upregulation of IL-36γ. IL-36γ mRNA and protein have been shown to be induced in keratinocytes following treatment with TNFα (Kumar et al., 2000). Consistent with findings in bronchial ECs (Chustz et al., 2011), IL-36γ levels were increased following TNFα treatment in 3-D FRT EC, demonstrating that expression of IL-36γ is inducible in the human FRT. TNFα and IL-36γ participate in a mutually reinforcing gene expression loop, initiated by IL-36γ, TNFα, as well as other cytokines (Friedrich et al., 2014). Increased levels of TNFα have been shown to result from microbial product exposure and pathogenic insults in the FRT (Jarvis et al., 1999; Agrawal et al., 2008; Hjelm et al., 2010; Radtke et al., 2012; Krzyzowska et al., 2013; Doerflinger et al., 2014) and as such, IL-36γ could be bolstering the inflammatory cascade in the FRT.

Poly (I:C), FSL-1, and flagellin treatments each resulted in unique IL-36γ expression and secretion patterns in our 3-D FRT EC models. TLR2, 3, 5, and 6 are the most abundantly expressed and functional in the FRT and our 3-D human FRT models (Herbst-Kralovetz et al., 2008; Hjelm et al., 2010; Radtke and Herbst-Kralovetz, 2012; Radtke et al., 2012). Flagellin targets TLR5, which activates the pro-inflammatory NF-κB pathway in parallel to apoptotic caspase cascades (Zeng et al., 2006). FSL-1 signals through TLR2/6, which also leads to the pro-inflammatory NF-κB pathway, but also triggers the MAPK pathway leading to possible apoptosis through MyD88 and caspase 8 (Aliprantis et al., 2000). Increased levels of IL-36γ were detected in 3-D FRT EC cell culture supernatant following FSL-1 treatment, but not following flagellin treatment. The mechanism of secretion is unclear as IL-36γ lacks a conventional signal sequence (Towne et al., 2011), and further work must be performed to determine IL-36γ secretion mechanisms in FRT EC. Poly (I:C) triggers TLR3 in the endosome, activating inflammation through the NF-κB pathway. Poly (I:C) has also been shown to induce FRT EC pro-inflammatory cytokine secretion, recruiting immune cells that activate cellular defense mechanisms, limiting viral replication until an adaptive immune response is generated (Schaefer et al., 2005; Herbst-Kralovetz and Pyles, 2006). It is unclear whether poly (I:C) induces IL-36γ secretion via a signaling sequence or via cell death, but it is possible that IL-36γ acts as an alarmin, alerting surrounding cells of danger (Lian et al., 2012).

In our study, levels of extracellular IL-36γ significantly increased and intracellular IL-36γ decreased following high dose treatments with poly (I:C). In contrast, flagellin treatment in FRT EC resulted in increased intracellular IL-36γ protein with corresponding supernatants containing low levels of extracellular IL-36γ. These results are similar to IL-36γ expression patterns observed in a study by Lian et al. in keratinocytes (Lian et al., 2012). These microbial product-specific induction and secretion relationships suggest that IL-36γ influences TLR dependent cellular defense responses unique to different forms of microbial attack. Further studies are required, but our data suggest that IL-36γ secretion may play a role in antimicrobial host defense and regulating tissue specific immune responses to infection in the FRT by amplifying the mucosal immune response.

IL-36γ activates the release of cytokines and chemokines from surrounding cells, leading to inflammatory host defense against invading pathogens. Prolonged inflammation in the FRT may also act as a warning sign of infection, indicating that IL-36γ may act as a marker for FRT disease or inflammation. A recent proteomics report demonstrated that cervicovaginal lavages collected from women with bacterial vaginosis exhibited elevated levels of IL-36α and IL-36 receptor antagonist relative to controls, further supporting a role for IL-36 family members in the FRT (Borgdorff et al., 2015). Both 3-D vaginal and endocervical EC responded to rIL-36γ treatment by upregulating gene expression of IL-6, IL-8, IL-1β, and TNFα. Each of these immune mediators has been found to be elevated in human cervicovaginal lavages collected from women with STI (Trifonova et al., 2007; Kyongo et al., 2015), as well as in the cell lines used in our 3-D FRT EC cultures following STI exposure (Herbst-Kralovetz et al., 2008; Hjelm et al., 2010; Radtke and Herbst-Kralovetz, 2012; McGowin et al., 2013). However, preliminary studies have not shown significant changes in TLR expression following rIL-36γ treatment (data not shown). We found that treating the 3-D FRT EC with rIL-36γ induced expression of IL-36R and cellular IL-36γ. A study by Friedrich et al. with keratinocytes defined this autocrine function as a self-amplifying loop involving IL-17 (Friedrich et al., 2014). IL-36γ has also been shown to induce CCL20, a Th17 cytokine, in lung fibroblasts due to asthma and COPD (Chustz et al., 2011) and in dermatoses via LL-37 (Li et al., 2014). rIL-36γ induced upregulation of IL-36γ and IL-36R mRNA as well as CCL20 mRNA and protein in 3-D FRT EC models suggesting the induction of an IL-36γ amplification loop following TLR activation. The increased secretion of CCL20 and its impact on Th17 cell recruitment and activation may play a role in host defense against FRT insult and is a future area of investigation.

AMP are present in the FRT during homeostasis and production is increased following infection in the FRT, as detected in cervicovaginal lavages (Novak et al., 2007; Keller et al., 2012; Madan et al., 2012; Pellett Madan et al., 2015). AMP induction by IL-36γ has been observed in keratinocytes and bronchial ECs (Chustz et al., 2011; Lian et al., 2012; Li et al., 2014). We investigated a panel of AMP known to be expressed in the lower FRT (Yarbrough et al., 2015) for induction by rIL-36γ exposure. Our data shows that each of these AMP (CCL20, HE4, SLPI, HBD-2, and HBD-4) were induced by treatment with rIL-36γ, further promoting inflammation in FRT EC in a dose-dependent fashion. CCL20, HBD-2, and HBD-4 upregulation was more robust in 3-D vaginal EC relative to cervical EC. Following pathological insult, serine protease inhibitors HE4 and SLPI have an inverse relationship, for example in bacterial vaginosis, HE4 is elevated, whereas SLPI is decreased (Mitchell et al., 2008). Interestingly, rIL-36γ treatment induced HE4 and SLPI expression in 3-D FRT models, similar to IL-1 treatment (Bingle et al., 2006). Further investigation is needed to determine if AMP induction by IL-36γ enhances host defense or results in damaging inflammation.

In the FRT, IL-36γ may function as a key regulator of mucosal inflammation. This is the first study to report the induction and regulation of IL-36γ, IL-36R, and related immune mediators in the human FRT following microbial product exposure. Both IL-36γ and IL-36R were induced by rIL-36γ in a dose dependent fashion, as well as a number of AMPs, pro-inflammatory cytokines, and chemokines in the FRT. The IL-36 family has been associated with the pathogenesis of several inflammatory diseases. Our data suggest that IL-36γ could play a critical role in initiating, promoting and sustaining epithelial-mediated inflammation to microbial products in the FRT. As such, future work will be aimed at studying the role of the IL-36 family in the context of the FRT in terms of host response to vaginal microbiota, STI pathogens, and other reproductive disorders.

## Author Contributions

MH-K contributed to the conception and design of the experiments. SW and AT acquired the data. SW, AT, and MH-K all contributed to the analysis and interpretation of the data, drafting the manuscript and revising it critically for intellectual content and approve of the final version of the manuscript.

## Conflict of Interest Statement

The authors declare that the research was conducted in the absence of any commercial or financial relationships that could be construed as a potential conflict of interest.

## Abbreviations

ECs: epithelial cells
FRT: female reproductive tract
FSL-1: fibroblast stimulating lipopeptide-1
IL-36: interleukin-36
IL-36R: IL-36 receptor
KSFM: keratinocyte serum-free medium
PBST: PBS-0.05%Tween-20
qRT-PCR: quantitative real-time polymerase chain reaction
rIL-36γ: recombinant IL-36γ
STI: sexually transmitted infection
