# Supplementary figures and images for: IL-36γ Augments Host Defense and Immune Responses in Human Female Reproductive Tract Epithelial Cells
Source: Front Microbiol. 2016 Jun 17;7:955. doi: 10.3389/fmicb.2016.00955 (PMC4911402; doi:10.3389/fmicb.2016.00955)

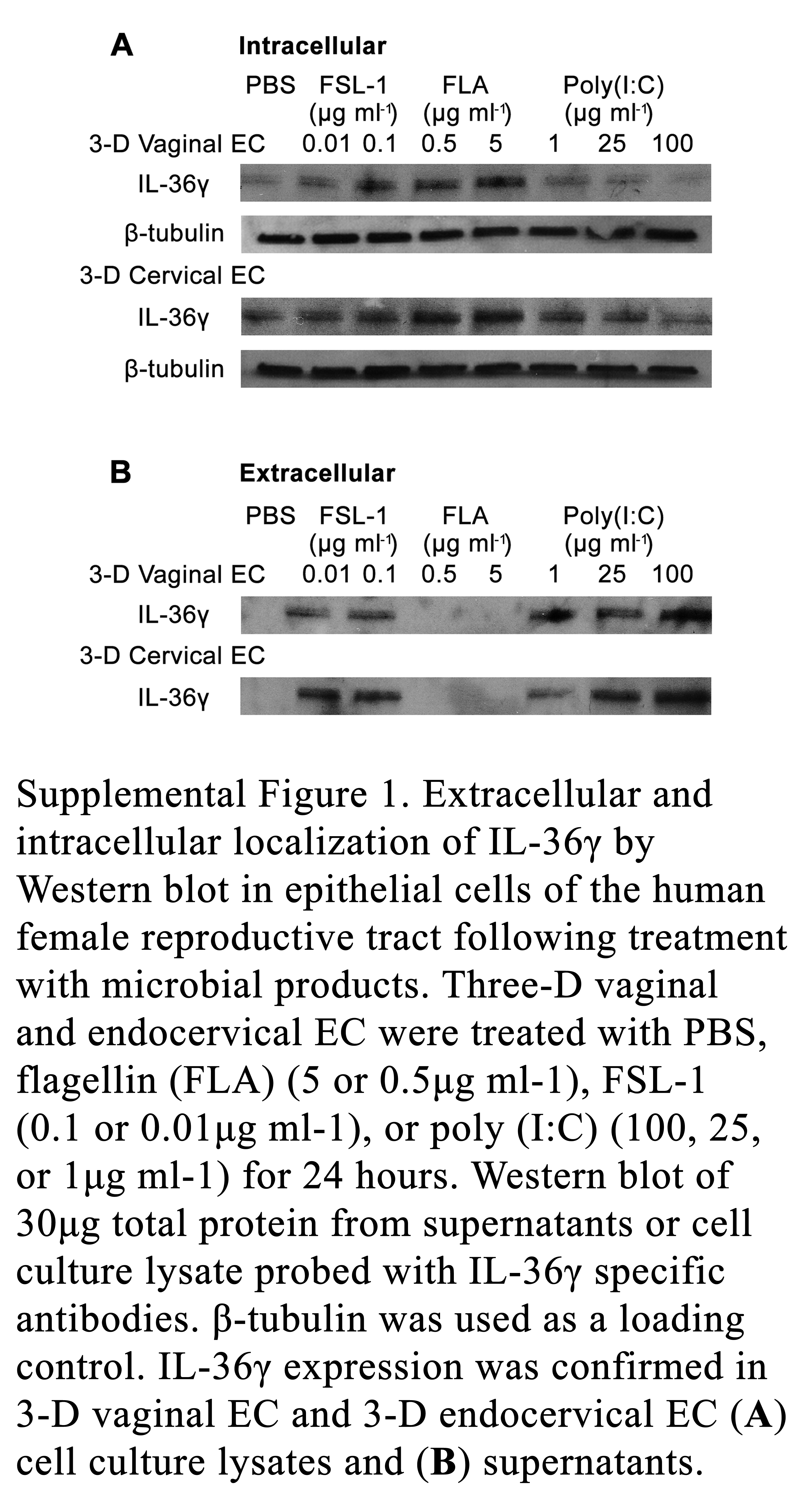

Supplement: Supplementary file 1 [file Image_1.TIF]
